# Type II Secretion Is Essential for Virulence of the Emerging Fish Pathogen, Hypervirulent *Aeromonas hydrophila*

**DOI:** 10.3389/fvets.2020.574113

**Published:** 2020-09-25

**Authors:** Priscilla C. Barger, Mark R. Liles, Joseph C. Newton

**Affiliations:** ^1^Department of Pathobiology, College of Veterinary Medicine, Auburn University, Auburn, AL, United States; ^2^Biological Sciences, College of Sciences and Math, Auburn University, Auburn, AL, United States

**Keywords:** virulent *Aeromonas hydrophila*, Type II secretion system, channel catfish, motile aeromonad septicemia, vAh pathogenicity, secreted proteins

## Abstract

Hypervirulent *Aeromonas hydrophila* (vAh) is an emerging pathogen in freshwater aquaculture systems. In the U.S.A., outbreaks of motile aeromonad septicemia associated with vAh result in the loss of over 3 million pounds of channel catfish from Southeastern production systems each year. *A. hydrophila* is a well-known opportunistic pathogen that secretes degradative and potentially toxigenic proteins, and the rapid mortality that occurs when catfish are challenged with vAh by intraperitoneal injection suggests that vAh-induced motile aeromonad septicemia may be, in part, a toxin-mediated disease. While vAh isolates from carp isolated in China possess complete Type I, Type II, and Type VI secretion systems, many of the US catfish isolates only possess complete Type I and Type II secretions systems. In order to determine the role of secreted proteins in vAh-induced disease, and to determine the extent of protein secretion by the Type II secretion pathway, an *exeD* secretin mutant was constructed using a recombineering method in the well-characterized US vAh strain, ML09-119. Wild-type and mutant secretomes were analyzed for protein content by SDS-PAGE and by assays for specific enzymes and toxins. Type II secretion-deficient mutants had a near complete loss of secreted proteins and enzyme/toxin activity, including hemolytic and proteolytic activity. The intact Type II secretion system was cloned and used to complement the deletion mutant, ML09-119 *exeD*, which restored protein secretion and the degradative and toxigenic potential. *In vivo* challenges in channel catfish resulted in complete attenuation of virulence in ML09-119 *exeD*, while the complemented mutant was observed to have restored virulence. These results indicate that secreted proteins are critical to vAh virulence, and that the Type II secretion system is the primary secretory pathway utilized for multiple effectors of vAh pathogenesis.

## Introduction

*A. hydrophila* is a ubiquitous Gram-negative bacterium capable of inhabiting a wide range of environments and acting as an opportunistic pathogen in fish, reptiles, amphibians, and mammals (1–3). In channel catfish production systems, *A. hydrophila* outbreaks in fish are common in spring and summer, particularly in Southeastern pond systems. Traditionally, *A. hydrophila* has been considered a secondary pathogen with low morbidity and mortality (4, 5). However, in 2009 a novel, highly-virulent strain of *A. hydrophila* was isolated from channel catfish during epidemic outbreaks of peracute motile aeromonad septicemia (MAS) in Alabama catfish production ponds (6). The bacterium continues to cause significant losses in the Southeastern catfish industry. This epidemic strain, referred to as hypervirulent *Aeromonas hydrophila* (vAh). vAh isolates within the sequence type 251 (ST251) pathotype are clonal in nature but show significant genetic variation from the typical, primarily opportunistic *A. hydrophila* (6, 7) and are capable of producing disease as a primary pathogen, resulting in rapid and devastating mortality in catfish production ponds (7, 8).

*A. hydrophila* are known to secrete numerus degradative and potentially toxigenic extracellular proteins and many possess multiple secretory mechanisms, including Type I, Type II, Type III, and Type VI secretion systems (9, 10). Type II secretion systems (T2SS) are found in myriad human, animal, and plant pathogens and Type II secretion (T2S) mediates a host of pathogenic processes, including host adhesion and invasion, host cell destruction, and tissue necrosis (11–13). While the role of T2SS in bacterial pathogenicity has been the primary focus of research, T2S is also crucial for survival in environmental species, as well as for opportunistic pathogens whose primary niche is outside the host (14).

While vAh is capable of causing disease as a primary pathogen it is, by and large, an environmental opportunist. Environmental niche manipulation is a hallmark strategy to increase fitness in generalist bacteria, and secretion of proteolytic, glycolytic, and metal-reducing molecules assist in bacterial habitat remodeling (15, 16). Bacterial influence over available habitat provides a competitive advantage; thus, bacteria possessing the capacity to release “remodeling” substances directly into the environment may be capable of rapid colonization of a self-created niche. T2S provides such a capability. By secretion of substrates that facilitate adhesion to biotic and abiotic surfaces, as well as proteins involved in biofilm formation, T2SS may support the shift between free-living and sessile lifestyles common in environmental bacteria and may increase niche exploitation capacity. Furthermore, T2SS substrates are generally highly stable in the environment, as evidenced by their presence in many extremophiles (17). While T2SS are widespread in *A. hydrophila* (10), the presence of a T3SS or T6SS has been generally considered more important for virulence (9) and, in some cases, essential to the infectious process (18, 19). Both T3SS and T6SS rely on needle-like “injectisomes” to mediate effector molecules directly from the bacterial cytoplasm into the target host cell (20, 21), while T2SS substrates are released from the periplasm through the outer membrane, in a seemingly indiscriminate manner, into the extracellular milieu (17). Interestingly, genomic comparisons of secretion systems in vAh strains found all 27 lacked a T3SS, and 18 of the 27 isolates, including all U.S. isolates of the ML09-119 pathotype, lacked a complete T6SS, though all vAh genomes contained at least three genes from the T6SS cluster (10). Remarkably, these remnant genes that code T6SS virulence-associated effector proteins, Hcp1 (hemolysin co-regulated protein) and VgrG (valine-glycine repeat G), appear to contribute to vAh pathogenicity. Despite the apparent lack of a functional T6SS, deletion of *hcp1* or *vgrG* genes from vAh ML09-119, the same strain used in these studies, resulted in a significant attenuation of virulence in catfish when challenged by immersion (10). These data suggest that these proteins may be exported by an alternative secretory mechanism in the vAh pathotype. The majority of Chinese vAh isolates possess a complete T6SS (10) and in these isolates the T6SS appears to play important roles in bacterial virulence, particularly for biofilm formation (22). In 2014, a second U.S. vAh pathovar containing a complete T6SS (S14-452) was isolated from diseased fish in Mississippi (23). This Mississippi vAh pathovar appears to be even more closely affiliated with the Chinese isolates than the vAh pathovar isolated from catfish in Alabama (7). However, lack of a complete T6SS in U.S. strains does not appear to reduce virulence in these strains, as mortalities in channel catfish immersion challenges were similar (~90%) in the vAh strains isolated from U.S. catfish that had partial (ML09-119) or complete (S14-452) T6SS components (7). Furthermore, when introduced by intraperitoneal injection, U.S. vAh ML09-119 was found to be more virulent in both channel catfish (*Ictalurus punctatus*) and grass carp (*Ctenopharyngodon idella*) compared to the Chinese vAh strain ZC1, which possesses both T2SS and T6SS (24). Collectively, these data indicate that secretion of many vAh toxins occurs via a secretory pathway distinct from T3SS or T6SS. In this study, a T2SS-deficient mutant was created and evaluated to determine the importance of vAh extracellular proteins (ECPs) secreted by T2SS to vAh virulence.

## Methods and Materials

### Bacterial Strains and Plasmids

Bacterial strains and plasmids used in this study are presented in Table 1. Hypervirulent *A. hydrophila* ML09-119 containing the recombinogenic plasmid, pMJH65 (25), was used as the wild-type vAh in which deletion mutations were generated.

**Table 1:** Primers and plasmids used in the construction and complementation of ML09-119 *exeD*.

**Bacterial Strains and Plasmids**	**Description**	**Source**
***Aeromonas hydrophila*** **strains**		
ML09-119	Wild-type, vAh, Isolated from diseased channel catfish in 2009	(6)
ML09-119 +pMJH65	Wild-type vAh carrying recombineering plasmid, pMJH65, Tet^r^	(25)
***E. coli*** **strains**		
SM10λpir	*thi thr leu tonA lacY supE recA*::RP4-2-Tc::Mu Kan^r^ *λpir*	(26)
HB101	F^−^*mcrB mrr* hsdS20(${\text{r}}_{{\text{B}}^{-}}$ ${\text{m}}_{{\text{B}}^{-}}$) *recA*13 *leuB6 ara*-14 proA2 lacY1 galK2 xyl-5 mtl-1 rpsL20(Sm^R^) glnV44 λ^−^	Liles Lab
E.cloni®10G	F- *mcrA* Δ(*mrr-hsd*RMS-*mcr*BC) *end*A1 *rec*A1 Φ80*dlac*ZΔM15 Δ*lac*X74 *ara*D139 Δ(*ara,leu*)7697 *gal*U *gal*K *rps*L *nup*G λ- *ton*A	Lucigen
BAC-Optimized Replicator v2.0®	F- *mcr*A Δ(*mrr*-*hsd*RMS-*mcr*BC) *end*A1 *rec*A1 Φ80d*lac*ZΔM15 Δ*lac*X74 *ara*D139 Δ(*ara,leu*)7697 *gal*U*gal*K *rps*L *nup*G (*att*L *ara*C-PBAD-*trf*A250 *bla att*R) λ-	Lucigen
**Plasmids**		
pKD3	Template plasmid for frt-flanked Cat cassette, Cat^r^	(27)
pMJH65	Conjugally transferable recombinogenic plasmid, Tet^r^	(25)
pCMT-flp	Temperature sensitive flp-recombinase, Tet^r^	(25)
pBAC-S	Non-self-transmissible expression vector, Apra^r^, Cam^r^	Varigen Biosciences
pRK2013	Helper plasmid for mobilization of non-self-transmissible plasmids, Kan^r^	Liles Lab

### Culture Media and Culture Conditions

Tryptic Soy Broth (TSB) (Bacto TSB, BD) and Luria Broth (LB) prepared according to manufacturer's directions was routinely used as the culture medium for growth of vAh and *Escherichia coli*, respectively, with the addition of 1.5% agar powder (Alfa Aesar) for solid culture. When necessary for selection, media were supplemented with chloramphenicol (Cam) (25 μg/ml), tetracycline (Tet) (10 μg/ml), colistin (Col) (10 μg/ml), kanamycin (Kan) (30 μg/ml), or combinations of those antibiotics. Super Optimal Broth (SOB) (Difco SOB, BD) was used for culture of electrocompetent cells and Super Optimal Broth with Catabolite Repression (SOC) was used as a recovery medium following electroporation. vAh strains were cultured at 30°C and *E. coli* strains were cultured at 37°C.

Bacterial strain vAh ML09-119 was removed from cryogenic storage and inoculated into 25 ml TSB media and grown overnight at 30°C with shaking. An aliquot of overnight culture was transferred to 70 ml of TSB and grown at 30°C on an orbital shaker at 180 rpm to mid-log phase, ~16 h.

### T2SS *exeD* Gene Deletion by Recombineering

#### Preparation of Antibiotic Cassette for Gene Replacement

Chloramphenicol was chosen as the selectable marker for gene deletion by recombineering. The pKD3 plasmid (27) was used as the donor for the chloramphenicol resistance gene, *cat*. The *cat* cassette, along with the flanking flippase recognition target (FRT) sequences, were extracted using appropriate primers (Table 2). To add homologous sequences required for recombineering, PCR was performed on the FRT*cat* cassette using FRT*cat*-specific primers with 60 and 65 base pair homology to upstream *exeC* and downstream *exeE* sequences, respectively (28). The PCR amplicon was purified using MicroElute Cycle-Pure kit (OMEGA Bio-Tek) following manufacturer's protocol. This purified DNA was used as the template for preparatory PCR using phosphorothioate primers. Following PCR, ten, 50 μl reactions were pooled, purified using ReliaPrep DNA Clean-up and Concentration system (Promega), and eluted in molecular-grade water to yield 590 ng/μl dsDNA. This *exeC*-FRT-*cat*-*exeE* linear dsDNA was then used as the selectable marker for *exeD* gene deletion.

**Table 2:** Oligonucleotides used in the construction and complementation of ML09-119 *exeD*.

**Oligonucleotide ID**	**Sequence (5^′^−3^′^)** *Phosphorothiote nucleotides	**Applications**	**Amplicon Size**
exeC-FRTCAT F	G*G*A *C*AG CTC TAC GAC GTT TAT GTC GGC TTG TCA GAA TAA TGA TTT GGA GTA GCA CCA AGA GTG TAG GCT GGA GCT GCT TC	Amplification of FRT-CAT sequence from pKD3 plasmid and addition of homologous sequence for recombineering	1,134 bp
exeE-FRTCAT R	C*A*G *C*TC GGG CAG GGC TGC CGG CAT GTC AAC GCC ATC CAG CTG GTA TGC CGC CAT TAC TTG CAT ATG AAT ATC CTC CTT A	Amplification of FRT-CAT sequence from pKD3 plasmid and addition of homologous sequence for recombineering	
exeC Homology F	G*G*A *C*AG CTC TAC GAC GTT TAT GTC GG	*exeD* amplification to check gene presence/deletions/insertions	2,037 bp (*exeD*) 1,134 bp (*exeD*:FRTcat) 2,550 bp (FRT scar)
exeE Homology R	C*A*G *C*TC GGG CAG GGC TGC C	*exeD* amplification to check gene presence/deletions/insertions	
exeE Gene F	ATG GCG GCA TAC CAG CTG G	RT-PCR	1,506 bp
exeE Gene R	TCA GTC TTC CCG GGT CAC GC	RT-PCR	

#### exeD Gene Deletion by Recombineering

Fifty microliter of freshly-prepared electrocompetent vAh Ml09-119 + pMJH65 was transferred to pre-chilled 1.5 ml tubes. One thousand five hundred nanogram exeC-FRTcat-exeE linear dsDNA (2.5 μl) was added to electrocompetent vAh ML09-119 cells, prepared as previously described (25), gently mixed, and transferred to chilled, 0.1 cm electroporation cuvettes. Cells were subjected to a single exponential decay pulse of 1.2 kV, 25 μF, and 200 Ω using an Eppendorf Eporator (Hamburg, Germany), followed by the immediate addition of 1 ml SOC recovery media, and incubated overnight with shaking at 30°C. Cells were then spread onto 2xYT agar plates supplemented with 25 μg/ml Cam and incubated at 30°C for up to 72 h to select for successful transformants.

To verify the deletion of *exeD* and the integration of the FRT*cat* cassette, PCR was performed on colonies that appeared within 72 h on Cam-selective media using primers with homology to *exeC* and *exeE*. Isolated colonies were carefully removed from the agar using sterile, disposable inoculating needles, resuspended in 20 μl sterile water, and 5 μl of this mixture was used as colony template. Wild-type ML09-119+pMJH65 served as the *exeD*+ control, and FRT*cat* cassette served as *Cat*+/*exeD*- control. Total reaction volume was 50 μl, with a final primer concentration of 20 pM. PCR was performed using EconoTaq Plus Green 2X Master Mix (Lucigen) in a Biorad T100 Thermal Cycler. PCR conditions included an initial denaturation step at 95°C for 3 min, followed by 34 cycles of denaturation at 95°C for 30 s, annealing at 55°C for 30 s, and extension at 72°C for 1 min. The final (35th) cycle included extension at 72°C for 5 min. Upon completion of PCR, 10 μl aliquots of each reaction were loaded onto a 0.8% agarose TAE gel containing 10 ng/μl ethidium bromide, and electrophoresed at 100 V for 1.5 h. Gel visualization and documentation was performed using the Gel Doc EZ Imager (BioRad) platform. To verify an in-frame deletion of *exeD*, RNA was isolated from vAh ML09-119 and *exeD*:FRT*cat* mutants and RT-PCR using *exeE*-specific primers was performed using Sunscript one-step RT-PCR kit (SYGNIS).

The pMJH65 plasmid, which confers Tet resistance, was cured from *exeD*:FRT*cat* mutants by heat induction as previously described (25). Following heat induction, cells were streaked for isolation on non-selective TSA plates, then isolated colonies were streaked onto TSA+Tet selective agar. pMJH65 plasmid loss was confirmed in isolated colonies by the inability to grow in the presence of Tet.

### Generation of T2SS ML09-119 *exeD* Markerless Mutant by Flippase (*flp*)-Mediated Recombination

Following successful generation of *exeD*:FRT*cat* mutants via recombineering, a markerless mutant, ML09-119 *exeD*, was generated by *flp*-mediated recombination using the *flp*-recombinase plasmid, pCMT-flp (25). The donor *E. coli* strain, SM10 λpir, was selected to mobilize pCMT-flp into vAh *exeD*:FRT*cat* via conjugation, as follows:

#### Isolation of pCMT-flp and Electroporation Into Mobilizable E. Coli SM10 λPir

The pCMT-flp plasmid, which confers Tet resistance, was purified from a common cloning strain of *E. coli*, E.cloni 10G (Lucigen), using E.Z.N.A. Plasmid DNA Mini Kit I (Omega Bio-Tek) following manufacturer's protocol, and was eluted at a final concentration of 11 ng/μl. Electrocompetent *E.coli* SM10 λpir were prepared as described above. pCMT-flp plasmid DNA (11 ng) was introduced into electrocompetent *E. coli* SM10 λpir via electroporation following the protocol above, but with a single pulse of 1.6 kV, 25 μF, and 200 Ω, and incubated at 37°C with shaking at 200 rpm for 4 h. Cells were then plated onto 2xYT+10 μg/ml Tet. Presence of the pCMT-flp plasmid was verified by the ability of colonies to grow on Tet-selective media.

#### Conjugal Transfer of pCMT-flp From E. Coli SM10 λPir to exeD:FRTcat

The *exeD*:FRT*cat* mutant was cultured in TSB+CAM at 30°C and *E.coli* SM10 λpir + pCMT-flp was cultured in LB+TET at 37°C with shaking to an OD_600_ = 1.0. One milliliter aliquots of donor and recipient cultures were transferred to separate sterile 1.5 ml microcentrifuge tubes, cells were pelleted by centrifugation at 10,000 *x g* for 5 min, and cell pellets were washed three times in antibiotic-free LB. After the final wash, cells were resuspended in 500 μl LB. To prepare the conjugation mixture, 125 μl of *E.coli* SM10 λpir + pCMT-flp was combined with 500 μl *exeD*:FRT*cat*, at a 1:4 donor: recipient ratio. Conjugation was performed using a spread-plate method, as follows. A 75 μl aliquot of the conjugation mixture was spread onto a blood agar plate, sealed with parafilm, and incubated overnight at 30°C. Plates were then divided into 8 sections, and 1/8 of the bacterial cells were removed from the agar surface using a disposable sterile inoculating loop and resuspended in 500 μl LB. Seventy-five microliter aliquots were spread onto selective LB plates containing 10 μg/ml Tet + 10 μg/ml Col, and plates were incubated at 30°C for up to 72 h. *exeD*:FRT*cat* transconjugants containing pCMT-flp were capable of growth in the presence of Tet.

#### Removal of Cat Gene From exeD:FRTcat by flp-Mediated Recombination

*flp*-mediated excision of the *cat* gene was initiated by heat induction as previously described (25). Briefly, transconjugant *exeD*:FRT*cat* + pCMT-flp colonies were removed from selective media and streaked for isolation onto TSA + Tet plates, and incubated at 30°C for 24 h. An isolated colony was then inoculated into TSB broth containing no antibiotic and grown at 30°C with shaking at 200 rpm to an OD_600_ = 1. Culture temperature was then increased to 37°C, and culture continued for 1 h to allow for flp-frt mediated gene deletion. The induced culture was then streaked for isolation on non-selective TSA and incubated at 30°C for 24 h. Isolated colonies were resuspended in 50 μl sterile water, and 5 μl of this template was used for colony PCR as described above. The colony template was also transferred to selective media containing Cam or Tet to confirm presence of pCMT+flp plasmid and the excision of *cat* gene. Isolated colonies that failed to grow in the presence of Cam and that revealed flp-deletion “scar” by PCR were confirmed as a ML09-119 *exeD* mutant. Prior to complementation, the pCMT-flp plasmid was cured from ML09-119 *exeD* mutants following the heat induction protocol outlined above. Successful loss of plasmid was confirmed by loss of Tet resistance in isolated colonies.

### Restoring T2SS by Whole-Pathway Complementation

To restore T2SS functionality in ML09-119 *exeD*, a whole-pathway complementation procedure was implemented.

#### Construction and Cloning of T2SS Vector

From an overnight culture of wild-type vAh ML09-119, high molecular weight genomic DNA (HMW gDNA) was isolated using MagAttract HMW DNA kit (Qiagen) following manufacturer's protocol. HMW gDNA was sent to Varigen Biosciences Corporation (Madison, WI), and CRISPR-Cas9 restriction was performed using guide RNAs specific to the T2SS genomic region. T2SS DNA was assembled into a linearized pBAC-S vector containing overlap sequence specific to the T2SS fragment and conferring resistance to Cam and apramycin. The T2SS vector (pBAC + T2SS) assembly was transformed into *E. coli* BAC-Optimized Replicator v2.0 electrocompetent cells and colonies recovered on selective media. Successful cloning was confirmed by colony PCR, *NotI* restriction digestion, and sequencing.

#### Complementation of T2SS by Tri-parental Mating

pBAC + T2SS was conjugally transferred from the donor strain (*E. coli* BAC-Optimized Replicator v2.0) to ML09-119 *exeD* by triparental mating, using the helper strain, *E. coli* HB101 pRK2013, to mobilize the pBAC + T2SS plasmid. An isolated colony of ML09-119 *exeD* was inoculated into TSB and grown at 30°C, with shaking at 200 rpm to an OD_600_ = 1.0. Donor and helper strains were inoculated into LB containing Cam and Kan, respectively, and grown at 37°C, with shaking at 200 rpm to an OD_600_ = 0.6. A 1 ml aliquot of ML09-119 *exeD* was pelleted by centrifugation at 13,000 rpm for 2 min at room temperature. Supernatant was removed and cells were resuspended in 1 ml LB and centrifuged as above. This wash step was repeated twice more. Following the final wash, ML09-119 *exeD* was resuspended in 500 μl LB. 1.5 ml aliquots of donor and helper strains were removed, pelleted, and washed as above. Following the final wash, donor and helper strains were resuspended in 250 μl LB. To prepare the conjugation mix, 62.5 μl donor and 62.5 μl helper cells were added to 500 μl ML09-119 *exeD* for a 1:1:4 donor:helper:recipient ratio. Hundred microliter aliquots were spread onto blood agar plates and incubated at 30°C for 24 h. Following incubation, cells were removed from 1/8 of the plate, as described above, and resuspended in 300 μl LB. Seventy-five microliter aliquots were spread onto selective LB plates supplemented with 10 μg/ml Col and 25 μg/ml Cam. Plates were incubated at 30°C for up to 72 h. Colonies present on selective media within 72 h were transferred to fresh LB + Cam + Col plates and streaked for isolation. An isolated colony was then selected and used as template for colony PCR. Transconjugant colonies that produced both *exeD* gene and *flp* scar amplicons were confirmed to have successful pBAC-S + T2SS vector integration, and were denoted *exeD*::T2SS.

### Determining Functional Role of T2SS

To determine the role of T2SS in secretion of putative virulence products, functional screening of wild-type and vAh T2SS mutants were performed. The necessity of T2SS for *in vivo* pathogenicity was measured in channel catfish host.

#### Growth Curves

To ensure cell viability in the T2SS mutant, growth curves were performed. Cells were diluted in TSB to an OD_600_ = 0.01, and 200 μl of cell suspension was placed in sterile, non-binding, 96-well polystyrene plates in quadruplicate. Plates were incubated at 30° C, with shaking, for 24 h in a Synergy HTX multi-mode reader, and readings were taken at OD_600_ every 30 min.

#### Secretome Preparation

To determine the role of T2SS in the secretion of putative virulence factors by vAh, secreted proteins of wild-type and T2SS mutants were isolated from planktonic cultures, as follows: vAh ML09-119 wild-type and all secretion mutants were cultured as described above. Cells were pelleted by centrifugation at 20,000 *x* g for 15 min at 4°C and the supernatant was decanted and retained. Cells were washed twice with cold, sterile PBS, pelleted as above, and the wash was added to supernatant. Remaining cells were removed by passage through a low-binding 0.22 μM vacuum filter (VWR). Cell-free supernatants were used as the starting point for secreted protein purification.

Secreted proteins were precipitated from cell-free supernatants by the addition of ammonium sulfate crystals (Fisher Scientific) to achieve 65% saturation, followed by incubation at 4°C on a rotary platform shaker with gentle mixing for 24 h. Precipitated proteins were collected by centrifugation at 30,000 *x* g for 45 min at 4°C, then dissolved in 10 ml cold Tris buffer (20 mM Tris-HCl, pH 7.6). Resuspended proteins were dialyzed twice, for 18 and 12 h, respectively, against the same buffer in 10 kDa dialysis cassettes [Slide-A-Lyzer (Thermo Fisher)]. After dialysis, the total volume was adjusted to 20 ml by the addition of cold Tris buffer. The protein concentration of each sample was determined by Bradford assay (Pierce Coomassie Plus Protein Assay, Thermo Fisher). These concentrated proteins were used for all assays.

#### PAGE Analysis

Secreted protein profiles were examined by polyacrylamide gel electrophoresis (PAGE). Aliquots of secreted proteins reduced with β-mercaptoethanol were loaded onto a pre-cast 12% Tri-glycine stain-free gel (NuSep) and electrophoresed at 200 V for 45 min. Gels were developed and documented using Gel Doc EZ imaging system (Bio-Rad).

#### Enzymatic Activity

*In vitro* activity of secreted proteins was measured using multiple substrates to determine degradative and toxigenic potential of secretomes.

#### Hemolysis

Hemolytic potential was measured using the method of Peatman et al. (29) with some modifications. In brief, heparinized blood from three channel catfish was pooled and diluted 1:10 in sterile phosphate buffered saline (PBS). A suitable dilution of protein in 150 μl PBS buffer was added to 25 μl diluted blood in sterile microcentrifuge tubes. Tubes were incubated at 30°C in an orbital shaker for 2 h. Positive control tubes representing 100% hemolysis contained 150 μl sterile distilled water and negative control tubes contained 150 μl sterile PBS in place of protein samples and were incubated with 25 μl diluted blood as above. Following incubation, tubes were centrifuged at 1,000*x g* to pellet unlysed cells and 150 μl of supernatant was transferred to 96-well flat bottom plates. Erythrocyte lysis was quantified by measuring hemoglobin absorbance at 415 nm in a multi-mode plate reader (Synergy HTX, Bio-Tek) and hemolysis was reported as percent of positive control.

#### Universal Protease Activity

Non-specific proteolytic activity was measured using HiLyteFluor 488-labeled casein as the substrate, following manufacturer's protocol with minor modifications (Sensolyte Green Fluorimetric Protease Assay Kit, AnaSpec, Inc.). Briefly, a suitable concentration of protein in 50 μl deionized water was added to triplicate wells of a black, flat-bottom 96-well plate with non-binding surface (Greiner Bio-One). Trypsin, diluted 50-fold in deionized water to a final concentration of 0.1 U/ml, acted as a positive control and sterile deionized water served as a substrate control. Following the addition of 50 μl labeled casein substrate, plates were mixed briefly and fluorescent intensity was measured at Ex/Em = 490 nm/520 nm every 5 min for 1 h in a multi-mode plate reader (Synergy HTX, Bio-Tek) with 30°C incubation temperature. Data were plotted as relative fluorescence units vs. time for each sample.

#### Elastase Activity

Elastase-specific activity was measured using 5-FAM/QXL^TM^ 520 labeled elastin as the substrate, following manufacturer's protocol with minor modifications (Sensolyte Green Fluorimetric Elastase Assay Kit, AnaSpec, Inc.). Briefly, a suitable concentration of protein in 50 μl deionized water was added to triplicate wells of a black, flat-bottom 96-well plate with non-binding surface. Elastase, diluted 50-fold in assay buffer to a final concentration of 8 μg/ml, acted as a positive control and sterile, deionized water was a substrate control. Following the addition of 50 μl labeled elastase substrate, plates were mixed briefly and fluorescent intensity was measured continuously at Ex/Em = 490 nm/520 nm, and data recorded every 5 min for 1 h in a multi-mode plate reader with 30°C incubation temperature. Data were plotted as relative fluorescence units vs. time for each sample.

#### *In vivo* Virulence

Channel catfish were challenged with wild-type and T2SS mutants to determine the role in *in vivo* virulence.

#### vAh Cell Preparation

Isolated colonies of wild-type vAh, ML09-119, and the T2SS-deficient mutant, ML09-119 *exeD*, were each inoculated into 20 ml of TSB. An isolated colony of T2SS complemented mutant, *exeD*::T2SS, was inoculated into 20 ml TSB + 25 μg/ml Cam. All isolates were incubated at 30°C with shaking at 200 rpm to an OD_600_ = 1.0. Ten milliliter of each culture was pelleted by centrifugation at 6,000 rpm for 15 min, and the supernatant was decanted. Cells were washed by resuspension in 10 ml sterile phosphate buffered saline (PBS), centrifuged as above, and cell washed decanted. Cells were then resuspended in 16 ml sterile PBS.

#### Catfish Challenges

Specific-pathogen free channel catfish, reared under Auburn University Institutional Animal Care and Use Committee (IACUC) maintenance protocol #2018-3251, were transferred to 30 gallon aquaria and allowed to acclimate for 1 week under flow-through conditions, with water temperature of 30°C, controlled by in-tank heaters. Air stones present in each tank provided aeration. Challenges were performed under the IACUC-approved guidelines described in IACUC #2020-3671 (Studies into the pathogenesis of virulent *Aeromonas hydrophila* in channel catfish). Each challenge group was comprised of ten fish per tank, with three experimental replicate tanks, for a total of 30 fish per challenge group. Challenge groups consisted of PBS injection control, wild-type vAh (ML09-119), T2SS-deficient mutant (ML09-119 *exeD*), and T2SS-complemented mutant (*exeD*::T2SS). Prior to challenge, fish were sedated by immersion in an anesthesia tank containing 80 mg/L tricaine methanosulfate (MS-222) buffered to neutrality with sodium bicarbonate. Sedation was characterized by slowing opercular movement and total loss of equilibrium. Sedated fish were challenged with 5 × 10^7^ CFU total cells in 200 μl PBS introduced by intraperitoneal injection. Control fish were injected with 200 μl sterile PBS. Fish were then returned to appropriate experimental replicate tank and monitored until fully recovered, as indicated by normal swimming and response to visual stimuli. Fish were monitored hourly for 8 h, then twice daily for 5 days, and moribund fish were euthanized by prolonged immersion in 300 mg/L neutral-buffered MS-222.

### Statistical Analyses

Statistical analyses were performed in Prism 8.2.0 (Graphpad). One-way ANOVA followed by Tukey's multiple comparisons post-test were performed on triplicate data with significance set at *p* < 0.05. Graphical representations of data were produced in Prism 8.2.0.

## Results

### Deletion of *exeD* Gene by Recombineering and Creation of Markerless Mutant

To determine the role of the T2SS in the virulence of vAh, the *exeD* gene, which codes for secretin, was targeted for deletion by recombineering. The three genes required for gene deletion via recombineering, *exo, bet*, and *gam*, were provided by the pMJH65 plasmid which had previously been introduced into vAh ML09-119 (25). Colony PCR verified the deletion of *exeD* (2037bp) and the incorporation of FRT*cat* (1134bp) (Supplementary Figure 1). In-frame deletion of *exeD* was confirmed by RT-PCR with *exeE* primers (data not shown) and growth curves were performed to verify cell viability following *exeD* deletion (Supplementary Figure 2). Positive *exeD* deletion colonies were denoted *exeD*:FRT*cat*. The pMJH65 plasmid was effectively cured from *exeD*:FRT*cat* by heat induction. The markerless mutant, ML09-119 *exeD*, wa*s* generated and colony PCR was performed and amplicons from colonies with successful gene deletion revealed the 193bp FRT “scar” (Supplementary Figure 3). Colonies that failed to grow in the presence of chloramphenicol and were confirmed by PCR were denoted ML09-119 *exeD*. The pCMT-flp plasmid was cured by heat induction, resulting in the loss of tetracycline resistance in the marker-less ML09-119 *exeD* mutant.

### Restoring T2SS by Whole-Pathway Complementation

The T2SS genomic region was cloned into a broad host-range bacterial artificial chromosome (BAC) vector pBAC-S and transformed into *E. coli* BacOpt v.2.0. Hundreds of transformants were recovered and successful cloning was confirmed by colony PCR and *NotI* restriction digest (data not shown). Conjugal transfer of pBAC-S containing the T2SS pathway into ML09-119 *exeD* was conducted and transconjugants were screened by PCR for the presence of *exeD* gene and *flp*-scar amplicons. Three colonies were selected for screening, and all three contained both *exeD* gene and *flp*-scar amplicons (Supplementary Figure 4). These colonies were denoted *exeD*::T2SS.

### Determining the Functional Role of T2SS

Cell growth curves were performed on WT and ML09-119 *exeD* to ensure cell viability in secretion-null mutants. While growth was slower, ML09-119 *exeD* was capable of reaching cell densities comparable to WT, with OD_600_ of 1.49 and 1.59, respectively, at 24 h. Cell densities of *exeD*::T2SS complemented mutants were slightly decreased after 24 h (OD_600_ = 1.32 and 1.37), but substantial growth occurred which confirmed cell viability (Supplementary Figure 2).

**Figure 1:**
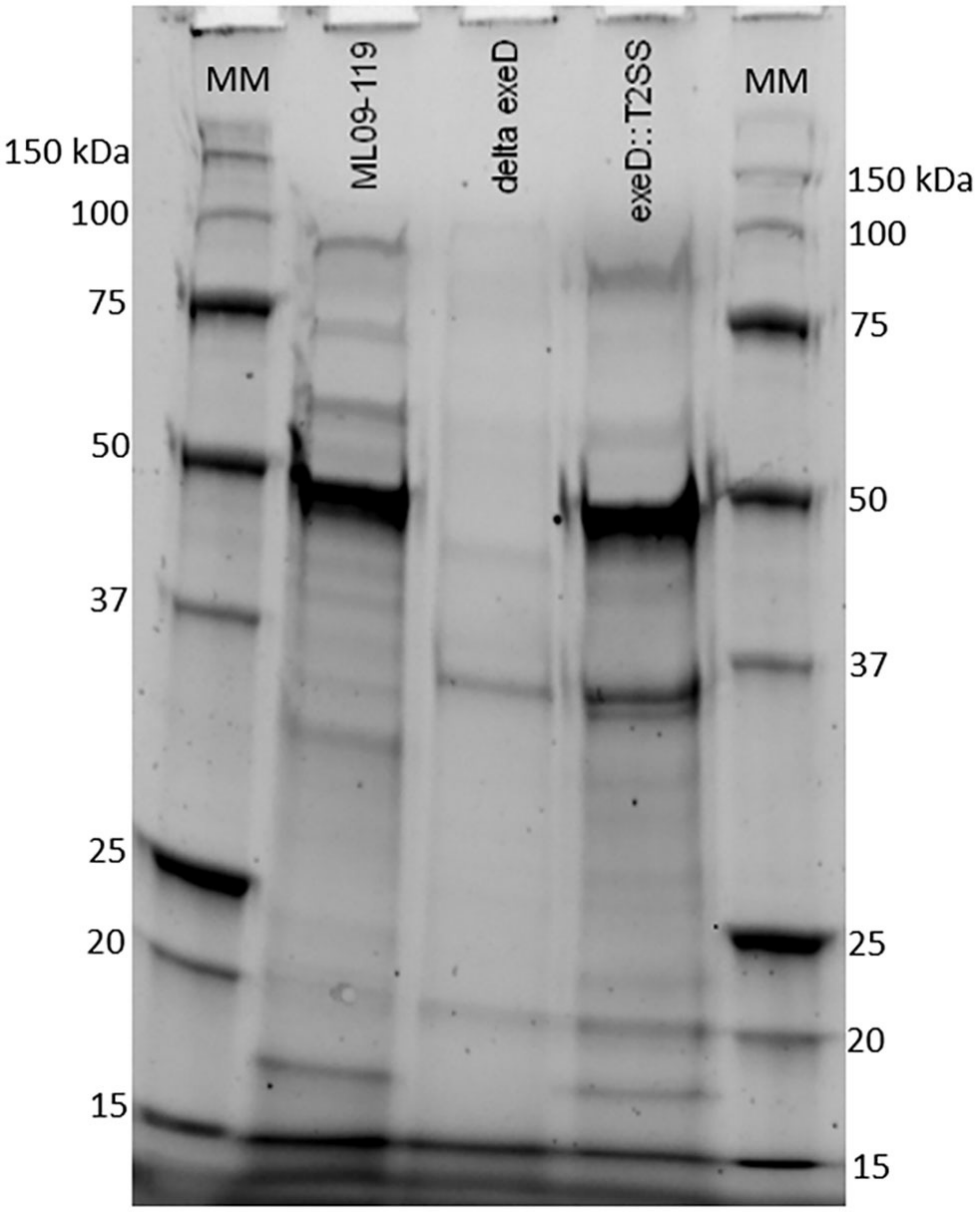
SDS-PAGE of proteins present in growth media of wild type ML09-119, ML09-119 *exeD* mutant, and T2SS-complemented mutant, *exeD*::T2SS. Minimal protein in the growth media of ML09-119 *exeD* is restored in the *exeD*::T2SS mutant, confirming the restoration of secretory function. MM, Molecular Marker.

**Figure 2:**
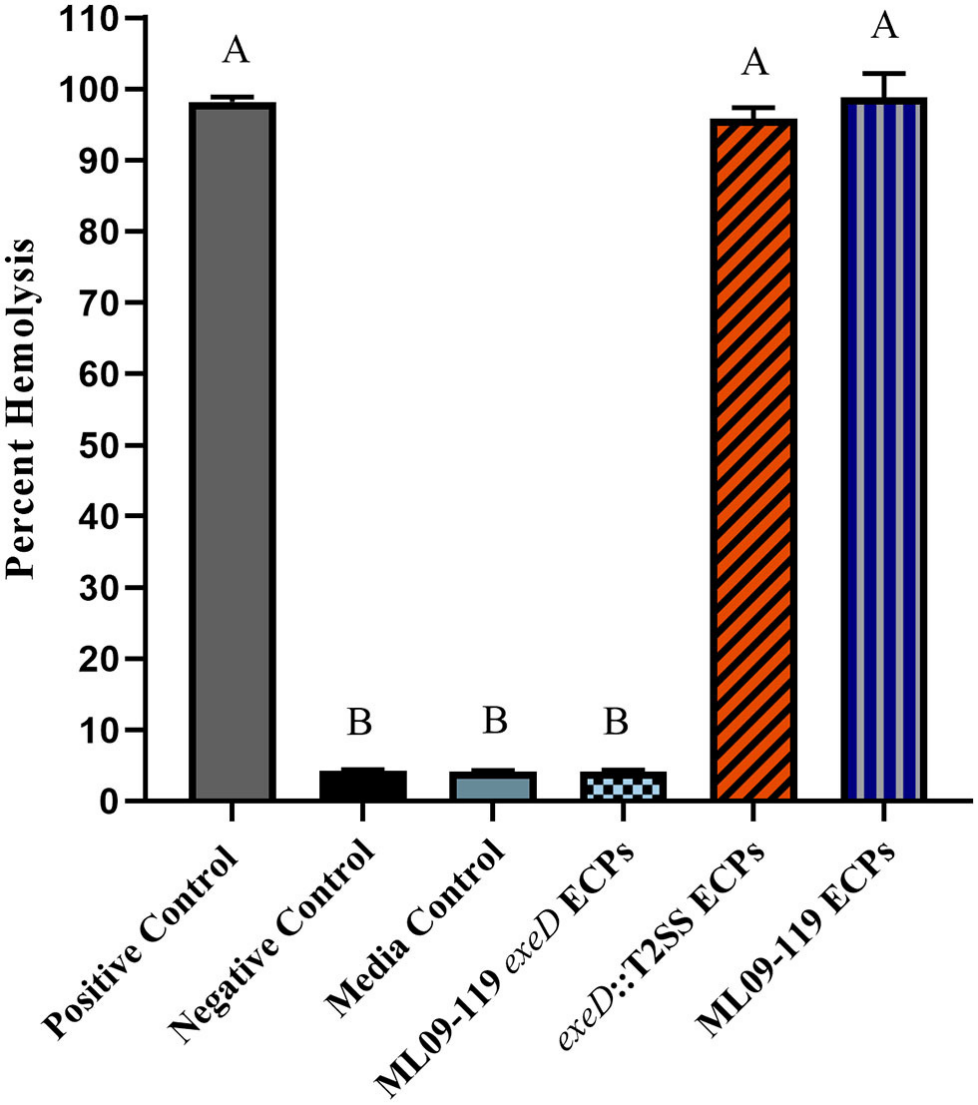
Hemolytic potential of wild type and mutant secretomes. Extracellular proteins (ECPs) from culture of each isolate were incubated with catfish erythrocytes for 2 h. Erythrocyte lysis was determined by measuring hemoglobin absorbance at 415 nm in multi-mode plate reader and hemolysis was reported as percent of positive control. All samples were assayed in triplicate. Statistical analysis consisted of one-way ANOVA followed by Tukey's multiple comparisons post-test with significance set at *p* < 0.05.

**Figure 3:**
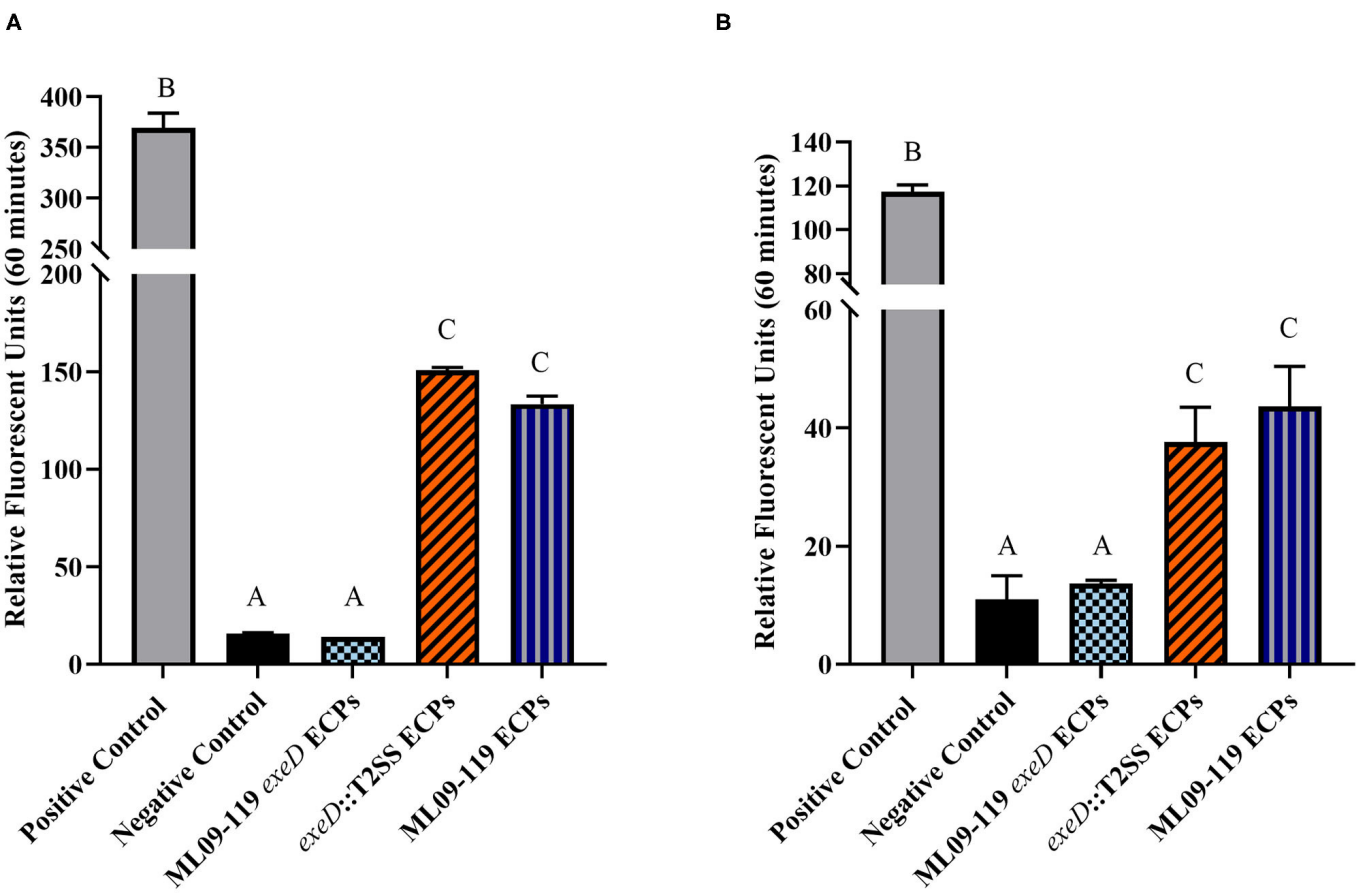
General proteolytic **(A)** and elastse-specific **(B)** degradative potential of vAh wild type and T2SS mutant proteins secreted under planktonic culture. The general proteolytic potential of secretomes was measured using HiLyteFluor 488-labeled casein as a substrate. The elastase-specific activity of secretomes was measure using 5-FAM/QXL^TM^ 520-labeled elastin as a substrate. Secreted protein from each isolate was incubated at 30°C with labeled casein or elastin and fluorescent intensity was measured at Ex/Em = 490/520 nm every 5 min for 1 h. Data from the final time point were plotted as relative fluorescence units vs. time for each sample. Degradative potential was lost completely in ML09-119 *exeD* mutants, but was restored in Δ*exeD*::T2SS complemented mutants. All samples were assayed in triplicate. Statistical analysis consisted of one-way ANOVA followed by Tukey's multiple comparisons post-test with significance set at *p* < 0.05.

To confirm that deletion of *exeD* resulted in a secretion-null mutant, and that T2SS complementation restored secretory function, secretomes of WT and mutants were prepared and visualized by PAGE (Figure 1). The ML09-119 *exeD* mutant secretome was nearly devoid of protein, confirming the loss of T2 secretion, while complementation with whole-pathway T2SS largely restored secretion. Major protein bands of ~90, 60, 48, and 18 kDa in the WT secretome were absent from ML09-119 *exeD*, but secretion was restored in *exeD*::T2SS. While complementation restored secretion, significant variability in WT and *exeD*::T2SS secretomes remained. Overall, the WT secretome appeared to be more complex than that of exeD::T2SS. One major band of ~35 kDa was present in both the ML09-119 *exeD* mutant as well as *exeD*::T2SS, but was not apparent in the WT secretome, suggesting that the protein(s) comprising this band may have had an alternate secretory mechanism. This variability is not unexpected, as maintenance and replication of plasmids can result in significant physiological changes in plasmid-containing cells (30). Plasmid presence in a cell often induce stress responses that mimic heat-shock response, resulting in the upregulation of multiple heat-shock proteins, decreases in cell growth rate, and changes in cell membrane (31, 32). Furthermore, plasmid maintenance and protein expression may contribute to the metabolic burden of the cells. Increases and decreases in protein expression of plasmid-bearing cells have been identified for a multitude of metabolic pathways, including protein biosynthesis, transcriptional regulation, carbohydrate metabolism, transport, motility, and energy metabolism (30), all of which would likely impact secreted protein profiles. Furthermore, the plasmid-borne T2SS may be under different regulatory expression which may result in changes in secreted protein expression. To determine if hemolytic and proteolytic proteins were secreted via T2SS, secretomes of WT and mutants were screened against multiple substrates. The hemolytic potential of planktonically-secreted proteins was measured using channel catfish erythrocytes as the target substrate. Mean percent hemolysis in WT secretomes was 98.8%. Hemolytic activity of ML09-119 *exeD* secretomes was 4.2%, identical to both negative and media control values, while complementation fully restored hemolytic activity in *exeD*::T2SS, resulting in mean percent hemolysis of 95.8% (Figure 2). The proteolytic potential of secretomes was measured against a casein substrate. No casein proteolysis was present in the supernatant of ML09-119 *exeD*, whereas complementation restored the caseinolytic potential in the *exeD*::T2SS mutant (Figure 3). Elastase-specific activity of secretomes was also measured, revealing no elastinolytic activity in the secretome of ML09-119 *exeD*, while complementation of TS22 restored elastinolytic activity (Figure 3).

**Figure 4:**
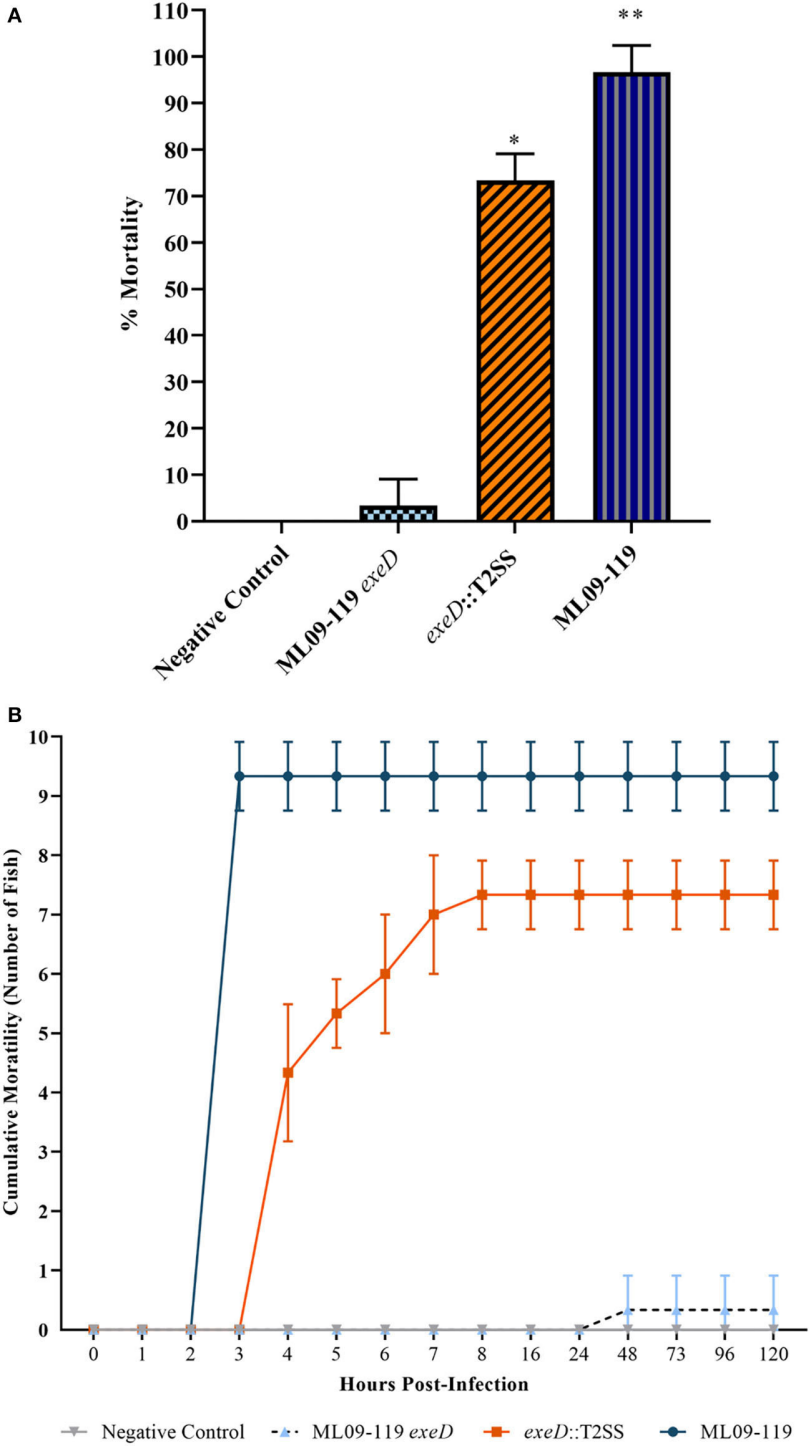
Percent channel catfish mortality **(A)** and mortality over time **(B)** when challenged with wild type and T2SS mutants. Each challenge group was comprised of ten fish per tank, with three experimental replicate tanks, for a total of 30 fish per challenge group. Fish were challenged with 200 μl PBS containing 5 × 10^7^ CFU of bacteria by intraperitoneal injection. Negative control fish were injected with 200 μl sterile PBS. Fish were contained in aquaria at 30°C. Wild type mortality was 96%, with death occurring in under 5 h. T2SS-complemented mutant, *exeD*::T2SS, mortality was 73%. Survival was 100% in the Negative Control group and 3% in T2SS deletion mutant, ML09-119 *exeD*. The complete loss of virulence seen in the ML09-119 *exeD* mutant was largely restored by T2SS complementation, supporting the hypothesis that T2SS and T2SS substrates play a significant role in the pathophysiology of vMAS. Statistical analysis consisted of one-way ANOVA followed by Tukey's multiple comparisons post-test with significance set at *p* < 0.05. * and ** represent statistically significant variability between challenge groups.

To determine the importance of T2SS and, thus, T2SS elaborated proteins in the pathogenicity of vAh, *in vivo* challenges were performed on channel catfish fingerlings. Catfish were challenged with 5 × 10^7^ CFU vAh WT and mutants by IP injection. Fish challenged with vAh WT were dead or moribund after 4 h, with average mortality of 96%. *exeD*::T2SS-challenged fish reached 50% mortality by 5 h and 73% mortality by 8 h, a 1.26-fold reduction in virulence from WT, while ML09-119 *exeD*-challenge produced 3% mortality after 5 days (Figure 4). Due to the peracute mortality in fish challenged by IP injection, external lesions that are commonly indicative of vMAS in naturally infected fish, including widespread petechiation, exopthalmia, and dermal necrosis (33), were largely absent. External gross examination revealed mild hyperemia at the base of fins in WT and *exeD*::T2SS-challenged fish. Few fish in these challenge groups also had a small (1–2 mm) area of hemorrhage in the skin and subjacent musculature at the injection site. Internally, a small amount of serosanguinous fluid was present in the abdominal cavity. Internal lesions included moderate hyperemia of the mesenteric tissues, mild to moderate swelling and congestion of the liver, and head and trunk kidneys. The spleen was ~3 times normal size, and bright red. There were no obvious differences in the pathology of WT and exeD::T2SS-challenged fish. No external or internal lesions, other than small foci of injection site hemorrhage in the skin, muscle, and peritoneal membrane were seen in ML09-119 *exeD* and control-challenged fish.

## Discussion

*A. hydrophila* strains are well-documented producers of putative virulence proteins. Historically, these proteins have been documented to target host cells by secretion through T3SS and/or T6SS (9, 18, 34, 35), while T2SS is considered a general secretion pathway, playing important roles in environmental organisms (14) and opportunistic pathogens (12). No vAh isolated from diseased catfish or carp has been found to possess a T3SS. While vAh strains isolated from carp in China, and some vAh strains isolated from the U.S. apparently have a complete and presumably functional T6SS (10, 23), many virulent vAh strains including ML09-119 lack a complete T6SS. While vAh do possess T1SS, as well as a tight adherence (Tad) secretin, primarily responsible for exporting Flp pili (36), these secretion pathways transport specific proteins and are likely not capable of the exportation of a vast array of proteins, particularly from within the periplasmic space. Therefore, the aim of this research was to study the role of T2SS and its effectors in the pathogenicity and virulence of vAh. To this end, a T2SS-deficient mutant was generated and was found to lack the vast majority of secreted proteins in its growth medium. Complementation of the T2SS restored the ability of the *exeD* mutant to secrete diverse proteins into the culture medium. While the secretome of *exeD*::T2SS appeared less complex than that of WT, multiple major protein bands were present in both WT and *exeD*::T2SS secretomes, including bands at ~90, 60, 48, and 18 kDa. While proteins cannot be identified based solely on PAGE analysis, previous research examining extracellular proteins of vAh reported the presence of at least 14 putative virulence proteins, including elastase, two hemolysins, lipase, and other potential toxins (37). Those findings were further supported by a second study (38) that reported catfish antibody production to vAh secreted proteins including chitinase, outer membrane proteins, metalloprotease, lipase, hemolysin, and elastase. In the latter study, Zhang et al. identified hemolysin and elastase within an ~60 kDa protein band, and chitinase and chitodextrinase within a 90 kDa band. This suggests that these bands in WT and *exeD*::T2SS could also contain hemolytic and degradative proteins. Furthermore, *in vitro* functional assays assessing the degradative and hemolytic potential of WT and mutant vAh found a complete loss of degradative capacity against casein and elastase in ML09-119 *exeD*. While vAh virulence is almost assuredly multi-factorial, the role of elastase in disease initiation and progression has been well documented. One study reported that the presence of elastase was required for *A. hydrophila* virulence in a rainbow trout (*Oncorhynchus mykiss*) model, with LD_50_ values two orders of magnitude higher in the elastase-deficient mutant (39). In *Pseudomonas aeruginosa*, elastase contributes to the invasiveness of the organism, likely by working in concert with other secreted proteins to weaken epithelial barrier function (40, 41). In *P. aeruginosa*, elastase is also responsible for significant tissue damage, especially in cystic fibrosis patients and those with other chronic respiratory infections. Elastin is critical for elasticity in lung tissue, and degradation by elastase has been implicated in lung fibrosis (41, 42). Elastase may also be capable of degradation of other structural proteins, such as collagen types III and IV, which may lead to the destruction of the basement membrane, granting bacterial ingress to the dermal tissue (39, 40). Elastase works in concert with other secreted proteins to increase tissue destruction and previous work in this lab identified a plethora of secreted proteins with degradative potential. The loss of general and elastase-specific degradation in ML09-119 *exeD* supports the hypothesis that T2SS is the main secretory network used by vAh ML09-119, and is likely responsible for secretion of a vast majority, if not all, degradative virulence proteins. In aquatic environments, degradative proteins act on natural substrates as a means of nutrient acquisition (43–45). Thus, while secreted proteins are likely of paramount importance for initial host colonization, the peracute nature of vAh-induced MAS (vMAS) mortality is most likely attributable to the array of hemolysins, enterotoxins, and cytotoxins produced by vAh. In order to test the degree of vAh toxin secretion through T2SS, secretomes of wild type and mutants were screened for hemolytic capacity. *A. hydrophila* hemolysins have been implicated as major virulence factors in fish and human *A. hydrophila* isolates and are often considered the main virulence factor of pathogenic *A. hydrophila* (9, 46, 47). *In vitro* hemolysin assays verify the hemolytic potential of vAh wild-type secretomes. In contrast, ML09-119 *exeD* secretomes had a complete loss of lytic potential (Figure 2), again, suggesting the vast majority of vAh hemolysins are secreted via T2SS. This is somewhat surprising, as *E. coli* and closely-related *V. cholerae* have been found to secrete hemolysins via the T1SS (48). vAh possess a functional T1SS and it would not have been unexpected for a certain degree of hemolytic activity to be retained by ML09-119 *exeD*. The restoration of proteolytic and hemolytic potential by whole-pathway T2SS complementation further supports the hypothesis that vAh requires T2SS for virulence.

*In vivo* challenges in catfish indicated that T2SS substrates are required for the development of vMAS and for vAh pathogenesis. Complete attenuation of virulence in ML09-119 *exeD* and restoration of virulence in *exeD*::T2SS provide convincing evidence that vMAS is, in large part, a toxin-mediated disease, and that T2SS and its substrates are required for vAh virulence. While virulence was not fully restored in *exeD*::T2SS, the apparent reduction in virulence in the complemented mutant was not completely unexpected. Previously identified decreases in growth rate, coupled with the removal of antibiotic selective pressure, which ensures plasmid maintenance and expression, were likely responsible. However, gross pathology of vMAS caused by WT and *exeD*::T2SS were indistinguishable, suggesting that the same virulence mechanisms are at play. Furthermore, while external lesions typically present in naturally-infected catfish were largely absent due to the peracute nature of vMAS in IP-injected fish, internal lesions were indicative of septicemia.

In conclusion, the experiments performed herein have demonstrated that T2SS and the T2SS elaborated effectors play a vital role in the pathogenicity of vAh, and in the development of vAh-induced MAS in channel catfish. Most importantly, these data have shown that T2S is essential for the secretion of proteolytic and hemolytic proteins, and that a functional T2SS is prerequisite for the development of vMAS.

## Data Availability Statement

The raw data supporting the conclusions of this article will be made available by the authors, without undue reservation.

## Ethics Statement

The animal study was reviewed and approved by Institutional Animal Care and Use Committee at Auburn University [IACUC #2020-3671 (Studies into the pathogenesis of virulent *Aeromonas hydrophila* in channel catfish)].

## Author Contributions

PB, ML, and JN conceived and designed the experiments and contributed to the writing and editing of the paper. PB and JN performed *in vivo* challenges. PB performed all other experiments and analyzed data. ML provided expertise and direction in molecular methods. JN provided expertise and direction in fish health and disease. All authors contributed to the article and approved the submitted version.

## Conflict of Interest

The authors declare that the research was conducted in the absence of any commercial or financial relationships that could be construed as a potential conflict of interest.
