# Supplementary material for: Type II Secretion Is Essential for Virulence of the Emerging Fish Pathogen, Hypervirulent Aeromonas hydrophila
Source: Front Vet Sci. 2020 Sep 25;7:574113. doi: 10.3389/fvets.2020.574113 (PMC7544816; doi:10.3389/fvets.2020.574113)
Supplement: Supplementary file 1 [file Table_1.DOCX]

51

1

1

41

1

1

31

1

1


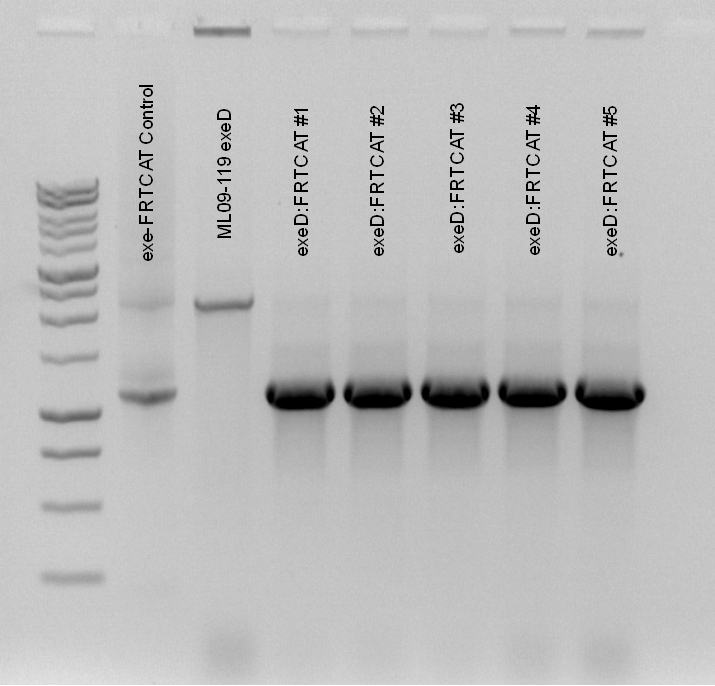


**10,000 bp**

**250**

**500**

**8000**

**6000**

**5000**

**4000**

**750**

**1500**

**2500**

**3000**

**2000**

**1000**

**MM**

11

1

1

21

1

1

**Supplementary Figure 1.** Confirmation of *exeD* deletion by homologous recombination in *exeD*:FRT*cat* mutants. Following electroporation of FRT*cat* cassette into vAH ML09-119 + pMJH65, colonies growing on selective media supplemented with CAM were selected and deletion of *exeD* gene (2037bp) was verified by colony PCR using primers that flanked *exeD*. Lane 1. Molecular Marker Lane 2. Positive Control, Lane 3. Negative Control, Lanes 4-5. Recombinant *exeD*:FRT*cat* mutants showing successful deletion of *exeD* and incorporation of FRT*cat* cassette (1134bp).


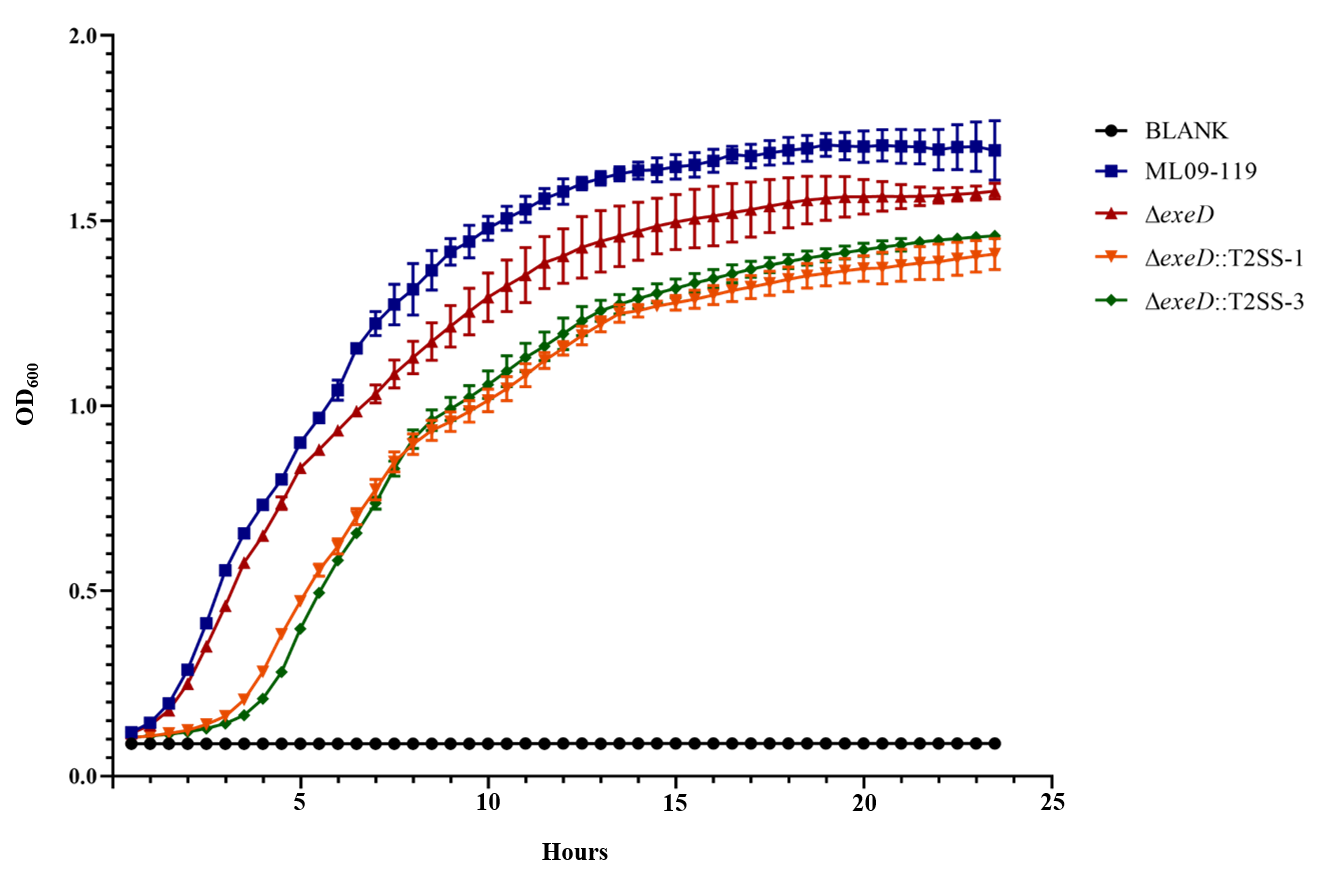


**Supplementary Figure 2.** 24-hour growth curve of WT ML09-119, ML09-119 *exeD* (Δ*exeD*), and *exeD*::T2SS (Δ*exeD*::T2SS-1 and -3) mutants. WT growth is more rapid, with a longer stationary phase. ML09-119 *exeD* growth is slower and cell density is slightly decreased. *exeD*::T2SS mutant growth rate is decreased, however, substantial growth occurs, confirming cell viability in all mutants.

A


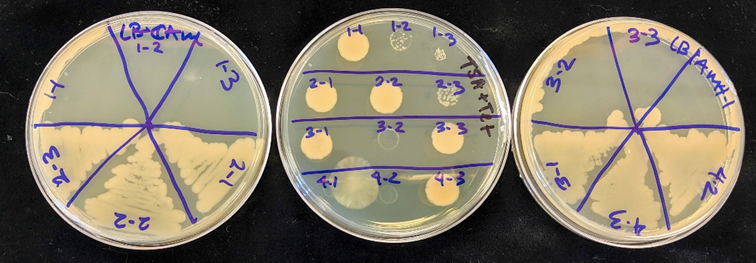


**2**

**3**

**4**

**5**

**6**

**7**

**8**

**9**

**10**

**11**

**12**

**13**

**14**

**15**

**16**

**17**

**18**

**19**

**1**


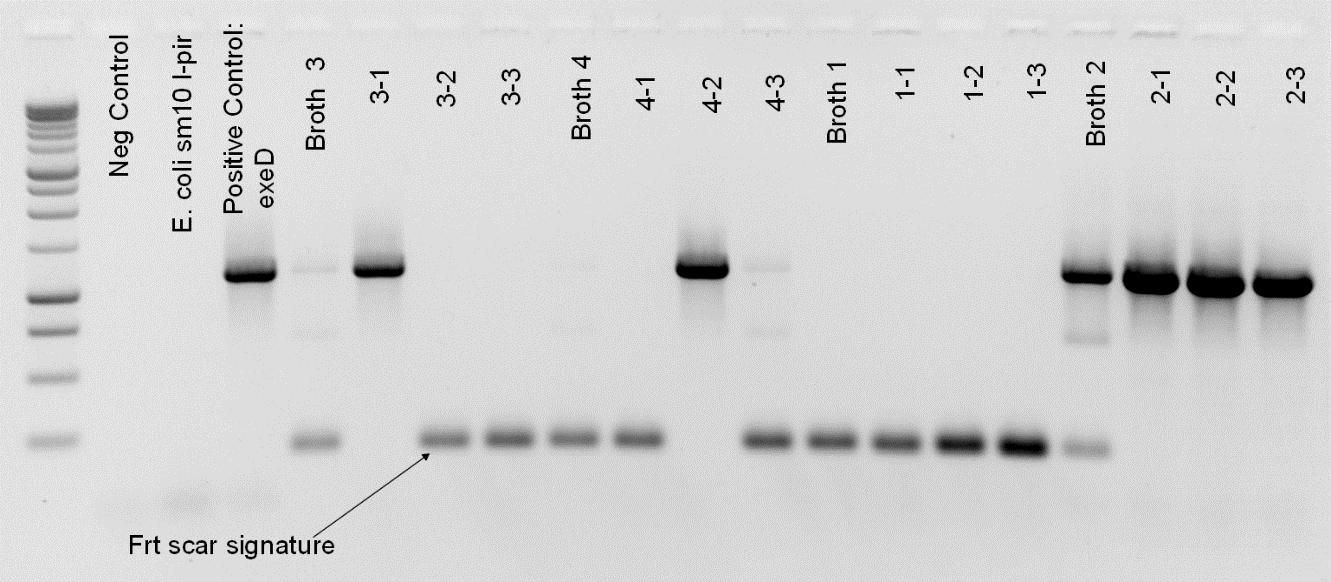


**10,000 bp**

**500**

MM

:FRT*cat*

**3000**

**2000**

**250**

**1000**

B

**Supplementary Figure 3.** Creation of ∆*exeD* mutant by FLP - mediated recombination. Following conjugal transfer of plasmid pCMT-flp into *exeD*:FRT*cat* mutant, flp-recombinase was heat induced for 1 hour to allow removal of *cat* cassette. pCMT-flp plasmid, which carried Tet resistance, was cured by continued growth at 37°C for 4 hours. Cultures were streaked for isolation on non-selective media, then isolated colonies were plated on media containing Tet and Cam (**A**). Colony PCR was performed on colonies that failed to grow on Cam, Tet, and Cam and Tet using primers flanking *exeD* (**B**). Colonies with amplicons showing the frt ‘scar’ signature (193bp), and that were unable to grow in the presence of Cam or Tet had successful FLP-mediated excision of *cat* gene and had successfully been cured of pCMT-flp plasmid. Lane 1. Molecular Marker, Lane 2. Negative Reagent Control, Lane 3. Negative Control, *E. coli* SM10 λpir, Lane 4. Positive Control, *exeD*:FRT*cat* mutant (1134bp), Lanes 5 – 20. *exeD*:FRT*cat*:*flp* colonies

**5**

**1**

**6**

**7**

**8**

**9**

**10**

**2**

**3**

**4**


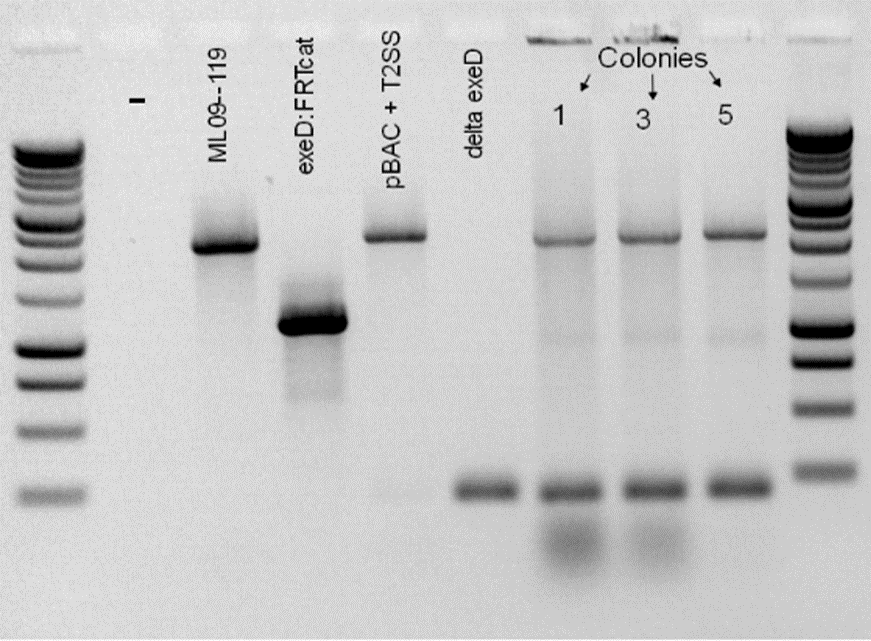


**10,000 bp**

MM

**250**

**500**

**750**

**1000**

**1500**

**2000**

**2500**

**3000**

Frt scar

*exeD*

**Supplementary Figure S4.** T2SS pathway complementation. Following tri-parental mating, transconjugants were recovered on selective media. Colony PCR was performed using *exeD*-specific primers to confirm the presence of the T2SS pathway. Successful complementation was verified by the presence of *exeD* gene and frt ‘scar’ amplicons. Lane 1. Molecular Marker, Lane 2. Negative Reagent Control, Lane 3. Positive Control (*exeD* gene amplified from wild type, 2037bp), Lane 4. Negative Gene Control (FRT*cat* insert amplified from *exeD*:FRT*cat* mutant, 1134bp), Lane 5. Positive pBAC Control (*exeD* gene amplified from the T2SS within pBAC), Lane 6. Frt scar Positive Control (amplification of ‘scar’ signature from ML09-119 *exeD*, 193bp), Lanes 7-9. *exeD*::T2SS transconjugants, Lane 10. Molecular Marker
